# An innovative circle drawing template in areolar tattoing

**DOI:** 10.1016/j.jpra.2023.07.004

**Published:** 2023-07-17

**Authors:** M. Faenza, A. Antonetti, E. Crisci, M.M. Nicoletti, G. Pieretti, G.A. Ferraro

**Affiliations:** a University of Campania Luigi Vanvitelli - Multidisciplinary Department of Medical Surgical and Dental Specialties - Plastic Surgery Unit, Naples, Italy; b University of Campania Luigi Vanvitelli School of Medicine and Surgery, Naples, Italy

**Keywords:** Breast reconstruction, Areolar reconstruction, Breast cancer

Dear Editor,

The final step in breast reconstruction is the recreation of the areola in order to restore the visual identity of a natural-looking breast.

An areolar reconstruction achieved by grafting skin or portion of the contralateral areola or dermal substitute is prone to additonal scars and umpredictable impariments of the pigmentation on long term.^1^, ^2^

Tattooing is the most common technique in areolar reconstruction and is usually performed at least 4–6 months after nipple reconstruction, when the projection of the reconstructed nipple has settled.

The color immediately after the procedure should be darker than desired, as it tends to fade within the first month.

Recent study demonstraed how a tattoo-only NAC reconstruction technique is an option to consider in selected patients with potential risk factors because reduces significantly the incidence of poor cosmetic outcomes after conventional two stage nipple areolar reconstruction^3^ .

In order to prevent the fading out of pigments it is crucial to deposit them in the intermediate papillary dermis.

In fact a placement of the tattoo ink in the upper dermis will be prone to vanish due to exfoliation of superficial dermal layers in 3–6 weeks, whereas a deep placement of pigments below the papillary dermis will stimulate the activity of macrophages leading to enzimatic degradation in few months.

Color, diameter and position of the new areola are chosen according to the contralateral breast in unilateral reconstructions or according to patient's preferences in bilateral breast reconstructions.

Many authors have described the use of disposable low-cost materials as dressings^4^ or surgical instruments.^5^

In this short communication we describe the use of an ostomy pouch baseplate as a template for areolar tattooing.

The main advantage is the presence of a cut-to-fit hole that can be customized by cutting the baseplate with curved scissors according to the desired diameter.

Moreover, the baseplate is adhesive and can therefore be applied directly to the patient, centering it on the previously reconstructed nipple.

Once this is done, the areolar tattoo is performed, obtaining both a natural shape and a precise position.

At the end of the procedure, the device is removed and a non-adherent gauze is placed onto the tattoo.

In this paper we described the use of an inexpensive and easily available device which on the one hand has a resistance that protects the patient from accidental passages of the tattooing handpiece and on the other has an adhesiveness that guarantees comfort for both the patient and the operator, leading them to mutual satisfaction (Fig. 1, Fig. 2, Fig. 3).

**Fig. 1:**
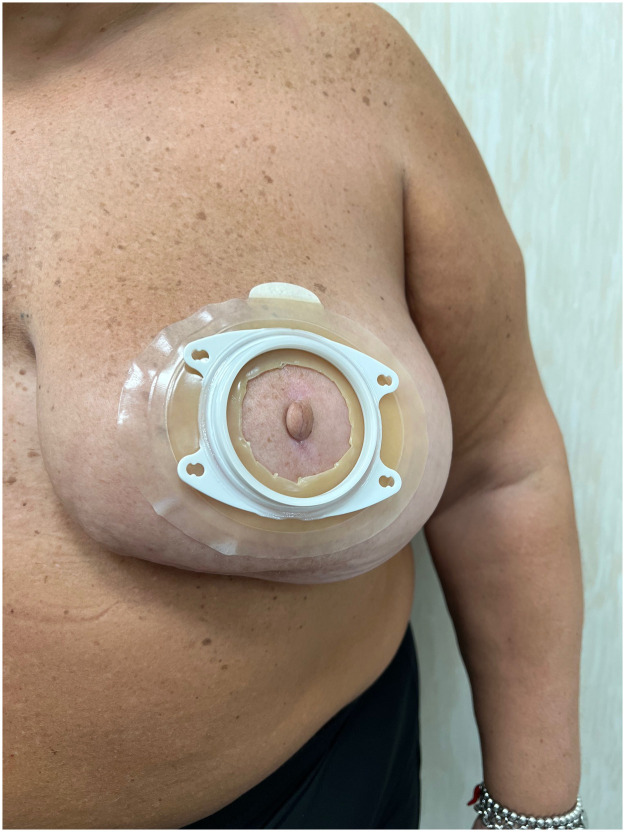
Preoperative assessment of diameter and position of new areola using the ostomy pouch baseplate.

**Fig. 2:**
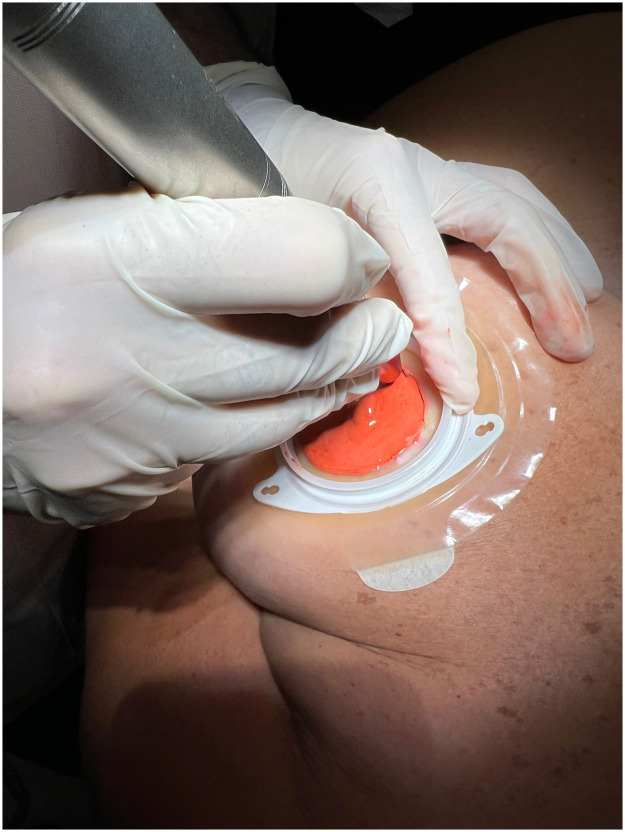
Intraoperative view of the areolar tattoo.

**Fig. 3:**
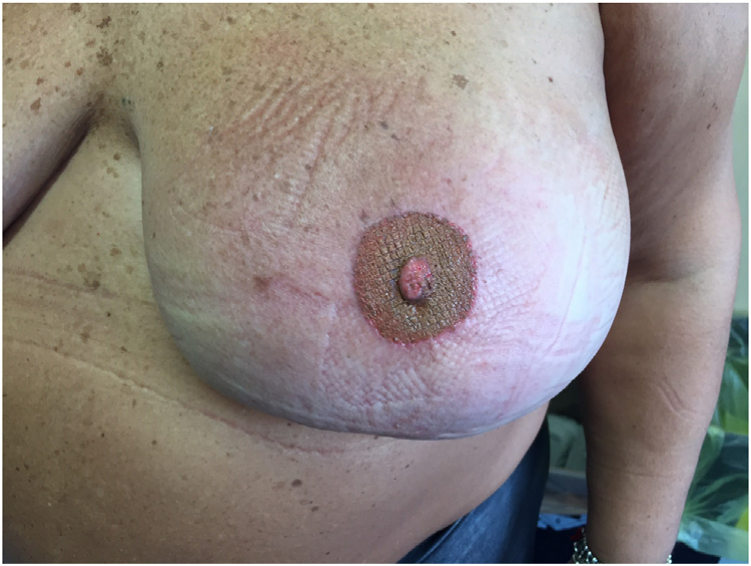
Postoperative appearence of the patient one month after the procedure with good cosmetic appearance of reconstructed areola.

## Ethical approval

Not required

## Funding

None.

## Declaration of Competing Interest

None.
